# Effects of Line Separation and Exploration on the Visual and Haptic Detection of Symmetry and Repetition

**DOI:** 10.1027/1618-3169/a000329

**Published:** 2016-10-18

**Authors:** Rebecca Lawson, Henna Ajvani, Stefano Cecchetto

**Affiliations:** ^1^ School of Psychology, University of Liverpool, UK

**Keywords:** regularity, bilateral symmetry, reflection, translation, object

## Abstract

**Abstract.** Detection of regularities (e.g., symmetry, repetition) can be used to investigate object and shape perception. Symmetry and nearby lines may both signal that one object is present, so moving lines apart may disrupt symmetry detection, while repetition may signal that multiple objects are present. Participants discriminated symmetrical/irregular and repeated/irregular pairs of lines. For vision, as predicted, increased line separation disrupted symmetry detection more than repetition detection. For haptics, symmetry and repetition detection were similarly disrupted by increased line separation; also, symmetry was easier to detect than repetition for one-handed exploration and for body midline-aligned stimuli, whereas symmetry was harder to detect than repetition with two-handed exploration of stimuli oriented across the body. These effects of exploration and stimulus orientation show the influence of modality-specific processing rather than properties of the external world on regularity detection. These processes may, in turn, provide insights into the nature of objectness in vision and in touch.

Regularities provide an important cue to the shape and structure of objects in our external world. Most research on regularities has focused on bilateral mirror reflection (henceforth termed symmetry). Symmetry is a property of many objects, including our own bodies and those of most animals, fruit, plants, and manmade objects such as tools (for reviews, see Treder, 2010; Tyler, 1995; Wagemans, 1995, 1997). Symmetry aids perceptual grouping, for example, by acting as a cue for figure-ground segregation (Machilsen, Pauwels, & Wagemans, 2009). Symmetry is usually easier to detect than other regularities such as repeated lines which have been translated (henceforth termed repetition) or rotational symmetry (Julesz, 1971).

It has been proposed that the presence of different types of regularity may be used to signal different properties in the world (Koning & Wagemans, 2009; Treder & van der Helm, 2007; Van der Helm & Treder, 2009). Of particular relevance for the present paper is whether symmetry may signal the presence of a single, bilaterally symmetric object, while repetition signals the presence of multiple, similarly shaped objects. This hypothesis has been supported by a number of studies which have reported an interaction between objectness and regularity-type (e.g., Baylis & Driver, 1995; Bertamini, 2010; Bertamini, Friedenberg, & Kubovy, 1997; Cecchetto & Lawson, 2016). The precise nature of this interaction varies across different studies (Koning & Wagemans, 2009) but, in general, symmetry is easier to detect for one-object stimuli with two regular sides than for two-objects stimuli where the facing sides of the two objects are regular. In contrast, repetition was easier to detect for two-objects stimuli than one-object stimuli.

Koning and Wagemans (2009) suggested that this interaction between objectness and regularity-type might reflect the basic strategies which vision uses to extract information, rather than high-level, cognitive strategies such as mental translations. To test their account, they again used pairs of edges belonging to either a single object or two objects. However, unlike previous studies which used 2D shapes their stimuli appeared to be planar, 3D objects tilted in depth by 45°. The use of these projected 3D objects to test regularity detection minimized figure-ground ambiguity and prevented the use of matching strategies involving simple mental translations. Despite these changes, Koning and Wagemans (2009) found an interaction between objectness and regularity-type, replicating previous results. They therefore concluded that structural differences between stimuli, and not the use of high-level matching strategies, underlay the one-object advantage for symmetry and the two-objects advantage for repetition.

However, all of the studies reviewed above tested vision only. Recently, Cecchetto and Lawson (2016) tested whether Koning and Wagemans’ (2009) conclusion generalized to regularity detection in a different modality, namely haptics (our sense of active touch). Haptics is the only other modality which is specialized at extracting shape information and there are many similarities in how vision and haptics identify objects. Across a number of studies, we have compared the ability of vision and haptics to do the same tasks using the same stimuli in order to examine whether effects found for visual processing generalize to haptics (e.g., Collier & Lawson, 2016; Craddock & Lawson, 2009a, 2009b; Lawson, 2009; Martinovic, Lawson, & Craddock, 2012). In the present study, we extended our approach to test regularity detection for symmetry and repetition. It is well established that haptics can detect symmetry (see Cattaneo et al., 2014, for a recent review) but, as far as we are aware, no other studies have investigated the haptic detection of repetition.

Cecchetto and Lawson (2016) found that there was an important difference between visual and haptic regularity detection. For vision, we found a one-object advantage for detecting symmetry and a two-objects advantage for detecting repetition, replicating the interaction reported by Koning and Wagemans (2009) and others. However, for haptics, there was a one-object advantage for both symmetry and repetition detection. These results suggest that effects on regularity detection may not be informing us about properties of the external world. Instead they may be telling us about differences in processing across our sensory systems. This alternative account was examined in the present study. However, in the present studies, unlike most previous studies including Cecchetto and Lawson (2016), we did not use planar, closed-contour shapes. As we now explain, this was due to a concern raised by Van der Helm and Treder (2009).

Van der Helm and Treder (2009) noted that most previous studies investigating the role of objectness on regularity detection tested anti-repetition rather than true repetition, see Figure 1. True regularities occur if two contours have the same polarities whereas anti-regularities occur if they have opposite polarities, for example with respect to curvature (so mismatched concavities and convexities), color, or luminance. Van der Helm and Treder’s (2009) findings indicated that the visual system treats anti-regularities differently to regularities. All of the studies discussed so far tested anti-repetition (Baylis & Driver, 1995; Bertamini, 2010; Bertamini et al., 1997; Koning & Wagemans, 2009; Cecchetto & Lawson, 2016). Van der Helm and Treder therefore argued that none of these studies actually tested whether repetition detection was easier for two-objects compared to one-object stimuli. They did, though, note that both Corballis and Roldan (1974) and Treder and van der Helm (2007) investigated this issue.

**Figure 1:**
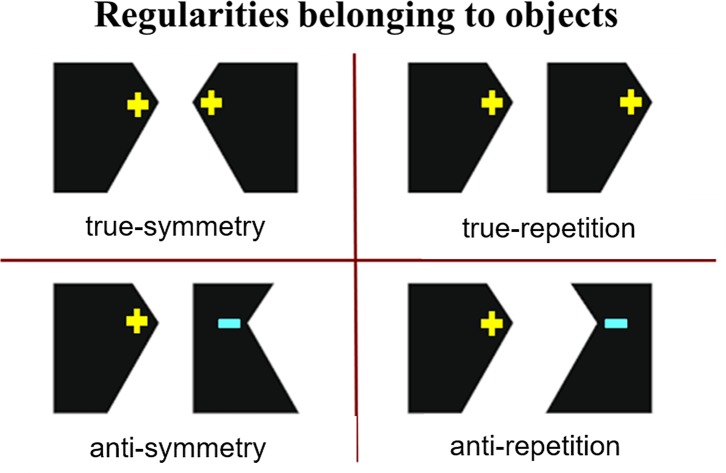
An illustration of four types of regular, two-objects, planar shapes varying in regularity-type (symmetry vs. repetition) and regularity-polarity (truly regular vs. anti-regular) based on Figure 1 of Van der Helm and Treder (2009). For anti-repetition and anti-symmetry stimuli, the two task-critical, regular contours have opposite polarities in terms of convexity (+) and concavity (−), defined with respect to the closed-contour object, and in terms of color and the luminance of the object, defined relative to its background. Baylis and Driver (1995), Bertamini (2010), Bertamini et al. (1997), Koning and Wagemans (2009), and Cecchetto and Lawson (2016) all used two-objects stimuli with the task-critical contours on facing sides of the two objects. This meant that these task-critical contours had true symmetry (the inner two lines in the top left case here) but anti-repetition (the inner two lines in the bottom right case here).

Corballis and Roldan (1974) asked people to compare dots in two 3 × 2 arrays. The two arrays were either adjacent (so they could be perceived as a single whole) or separated by a gap (so they may have appeared as two, separate objects). The dot patterns were either symmetrical or repeated so all the stimuli were regular and, unusually, the task was to discriminate symmetry from repetition. Symmetry was detected faster for adjacent compared to separated arrays, though this difference was not tested statistically. There was also a trend in the opposite direction for repetition detection (though it was probably not significant), so for an advantage for the separated arrays.

Treder and van der Helm (2007) used stereoscopic depth to assign the two halves of symmetrical and repeated dot patterns to either the same or to two different depth planes. They took advantage of the fact that location in depth influences the grouping of parts with nearby parts being more likely to be perceived as belonging to the same object. Splitting the stimuli across different depth planes disrupted symmetry detection but had little effect on repetition detection, so only symmetry processing clearly benefitted from structural correspondences occurring within a depth plane.

To summarize, Van der Helm and Treder (2009) argued that only two studies have investigated the interaction between regularity-type and objectness: Corballis and Roldan (1974) and Treder and van der Helm (2007). However, in both of these studies the interaction (symmetry detection being easier for one-object compared to two-objects stimuli, and vice versa for repetition detection) was found only for dot stimuli, and in neither study was there a clear two-objects advantage for repetition. In addition, Corballis and Roldan (1974) tested regularity discrimination rather than regularity detection, and they did not statistically test whether there was a one-object advantage for symmetry, or whether there was a two-objects advantage for repetition. Thus, there is still a dearth of evidence as to whether true repetition (as opposed to anti-repetition) is easier to detect visually for two-objects stimuli relative to one-object stimuli and this has never been tested for haptics.

This issue is of wider importance because it may provide insights into what defines an object in vision and touch. The concept of objectness is central to many aspects of spatial and conceptual organization in both perception and cognition. However, it has proven difficult to define what constitutes an object (Feldman, 2003). Researchers claiming to manipulate objectness often make little attempt to justify their choice of stimuli. The present study aims to introduce an approach which allows us to identify and to compare potential cues to objectness in both vision and touch. We do not assume that objectness is an all-or-nothing property of a stimulus and we think that multiple cues combine to determine whether a given stimulus is perceived as an object. We are not aware of any previous research that has tried to define what it means to be a haptic object. Our approach is therefore preliminary and we will not claim to provide conclusive evidence about the nature of objectness in haptics. Nevertheless, this topic is an important one which has been neglected for too long, and we think that progress can be made in trying to understand objectness across different modalities.

The present study tested a novel prediction based on previous research suggesting that symmetry detection is easier for one-object stimuli while repetition detection is easier for two-objects stimuli. We hypothesized that, on average, closer lines are more likely to be perceived as belonging to the same object and more distant lines as belonging to two different objects (see also Corballis & Roldan, 1974; Treder & van der Helm, 2007). This hypothesis leads to the prediction that it should be easier to detect symmetry when lines are closer because both cues (line separation and the type of regularity) indicate that one object is present. Conversely, repetition should be harder to detect when lines are closer because one cue (line separation) indicates that one object is present whereas the other cue (the type of regularity occurring – here, repetition) indicates that multiple objects are present. We investigated these predictions by testing whether effects of the type of regularity being detected (symmetry vs. repetition) interacted with line separation. This approach is conceptually similar to that taken by Treder and van der Helm (2007). They varied regularity-type (symmetry vs. repetition) and stereoscopic depth (the two stimulus halves were on the same vs. on different depth planes) to investigate how regularity detection was influenced by whether these cues provided consistent or conflicting interpretations of objectness. To avoid the issues discussed above arising from using anti-regularities (see Van der Helm & Treder, 2009), and to simplify the stimuli, the experiments reported here presented only lines rather than planar shapes (see Figure 2).

**Figure 2:**
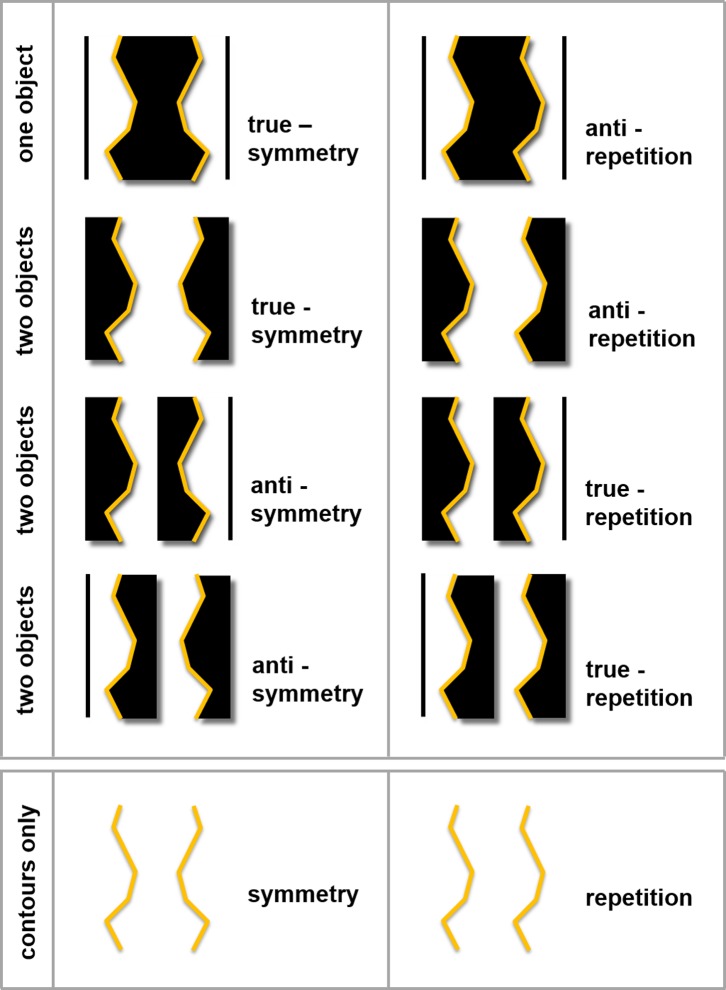
The upper box illustrates the type of planar, 2D stimuli that have previously been used to test the interaction between regularity-type (symmetry vs. repetition) and objectness (one vs. two). The pairs of task-critical, regular lines are highlighted here but they were not shown to participants. Baylis and Driver (1995), Bertamini (2010), Bertamini et al. (1997), Koning and Wagemans (2009), and Cecchetto and Lawson (2016) presented stimuli like those in the top two rows of the upper box (so true symmetry and anti-repetition stimuli); they did not show any true repetition or anti-symmetry stimuli. The lower box illustrates the line-only stimuli used in the present studies. Note that the task-critical, symmetrical, and repeated lines for all four rows of planar stimuli shown in the upper box are identical to these lines. Regularity polarity (true versus anti) cannot be defined unambiguously for the line stimuli since this would require labelling one side of the line as “inside”.

In summary, in the three studies reported here we contrasted how potential cues to objectness, such as the spatial separation between two lines, influenced the detection of symmetry and repetition. As in Cecchetto and Lawson (2016), we used matched stimuli and tasks to compare regularity detection for vision (Experiment 1) and for haptics (Experiments 2 and 3). The goal of this research was to investigate whether there is a one-object advantage (cued by a small line separation) for symmetry detection and a two-objects advantage (cued by a large line separation) for detecting true repetition, and whether any such effects found for regularity detection reflect modality-specific processing, or if they reveal differences arising directly from the presence of regularities out in the physical world.

## Experiment 1

In Experiment 1 participants saw pairs of vertically aligned, 2D lines. We investigated whether people found it harder to visually detect regularities (either symmetry or repetition, in separate blocks) when the horizontal separation between the two lines increased from 25 mm up to 50 mm and to 100 mm. We expected that smaller separations would make it more likely that the lines were perceived as belonging to a single object, whereas larger separations were more likely to be perceived as belonging to two different objects. We also hypothesized that symmetry is used as a cue for the presence of a single object, whereas repetition provides evidence for the presence of multiple objects. We therefore predicted that symmetry detection should be easier for small relative to large line separations. Here, nearby pairs of symmetrical lines provide consistent cues that a single object is present whereas distant pairs of symmetrical lines provide conflicting cues about objectness. The opposite pattern was predicted for repetition. Here, well-separated pairs of repeated lines provide consistent evidence for the presence of two objects, while nearby, repeated lines provide conflicting cues about objectness. However, there are independent reasons why regularity detection may be harder at large line separations, such as the difficulty of visually perceiving more peripheral stimuli. Any such effects would counter the expected large-separation advantage for repetition (while enhancing the predicted large-separation cost for symmetry). We therefore simply predicted that increasing line separation would disrupt symmetry detection more than repetition detection.

### Method

#### Participants

Twenty-four students from the University of Liverpool (16 females, mean age = 20 years, *SD* = 2.8, range 18–31) volunteered to take part in the experiment. In all of the experiments reported in this paper the participants had normal or corrected-to-normal vision, they self-reported as right-handed, they had no known conditions affecting their sense of touch, and most received course credits in exchange for their time. All the experiments received ethical approval from the local Ethics Committee.

#### Materials

**Figure 3:**
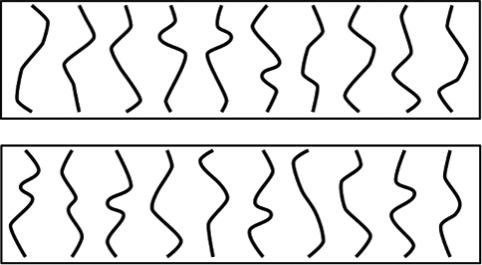
The 20 unique lines used to generate the regular stimuli for Experiments 1, 2, and 3. In Experiment 1 only, 20 additional unique lines were used which were produced in a similar way. The unique lines are shown here ordered from easiest (top left) to hardest (bottom right) in terms of accuracy in previous regularity detection tasks (discriminating symmetrical from irregular stimuli and repeated from irregular stimuli in Experiment 1 of Cecchetto & Lawson, 2016).

We produced a set of 480 pairs of lines based on the 40 unique lines used in Experiment 1 of Cecchetto and Lawson (2016). However, the vertices of these unique lines were rounded to ensure that when the lines were felt (in Experiments 2 and 3) there would be no sharp corners which might be difficult to explore by touch. Each unique line had four vertices with the top and bottom of each line vertically aligned, see Figure 3. Each unique line was paired with a mirror-reflected version of itself, with the same version of itself, with a mirror-reflected version of a different unique line and, finally, with a repeated version of a different unique line. This produced the symmetrical, repeated, and two irregular stimuli, respectively. For the irregular stimuli, unique line 17 could be paired with unique line 3 for its two irregular stimuli, while unique line 3 could be paired with unique line 8, and so on. There were 480 trials in total (40 unique lines × Regular/irregular stimuli × Symmetry/repetition regularity-type × Small/medium/large line separations).^1^

Pairs of lines were presented as 2D images on a computer monitor and were viewed from a distance of approximately 50 cm. The LCD widescreen monitor (Dell Inc., USA) was 58 cm diagonally and had a resolution of 1280 × 1024 pixels. Each line was 3 mm wide and 100 mm high. The top and bottom of each line was positioned 12.5 mm each side of the midpoint of the monitor for the 25 mm separated lines, 25 mm each side of it for the 50 mm separated lines, and 50 mm each side of it for the 100 mm separated lines. The 100 mm separated lines subtended around 11° × 11°.

#### Design

All participants did one block of symmetry detection and one block of repetition detection, with block order counterbalanced across participants. Each block had 240 trials (40 unique lines × Regular/irregular stimuli × Small/medium/large line separations). These trials were presented in a different, random order for each participant.

#### Procedure

**Figure 4:**
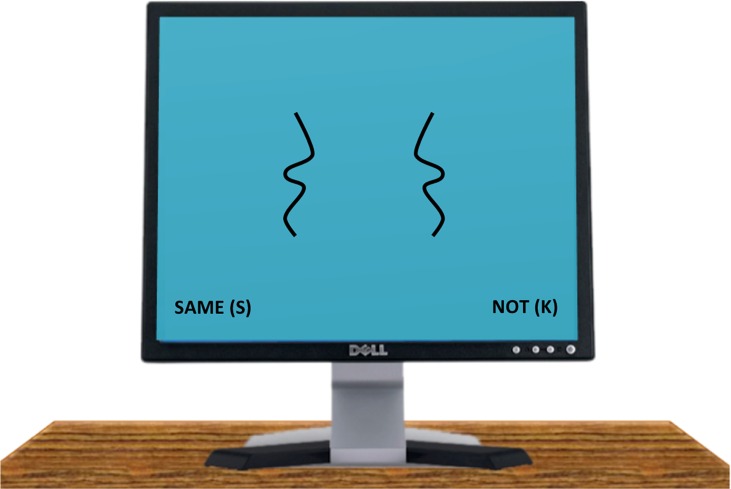
An example of a large line separation, symmetrical stimulus presented visually on the computer monitor in Experiment 1.

Participants sat in a normally lit room. Participants were instructed to center their body midline to the center of the computer monitor. Before starting each block, participants were told about the nature of the regularity they were about to detect, its orientation, and that the stimuli could have different line separations. Each block of experimental trials was preceded by 10 practice trials taken from that block. These practice trials were the same for all participants and they included five regular and five irregular trials and a mix of the three line separations. At the start of each trial, a central fixation cross appeared on the monitor for 1 s. This was replaced by the stimulus which remained on the monitor until the participant responded. Visual prompts about how to respond were presented on the monitor whenever the stimulus was visible, see Figure 4. Participants responded using the computer keyboard, pressing “s” for regular trials and “k” for irregular trials as quickly and as accurately as possible. Reaction times (RT) were recorded from stimulus onset until the participant responded. The experiment took around 30 min to complete.

### Results

Correct RT faster than 0.4 s or slower than 3.5 s were removed as outliers (less than 2% of trials).^2^ To be consistent with reporting in previous studies, ANOVAs were conducted on the mean correct RT and on the percentage of errors for regular trials only (also performance on irregular trials is difficult to interpret theoretically). In all three experiments reported here, we also analyzed measures of sensitivity (d′) and bias (c′) which included data from irregular trials, see Appendix. There were two within-participants factors in the ANOVAs: regularity-type (symmetry or repetition) and line separation (small, medium, or large).

Regularity-type was significant for RT, *F*(1, 23) = 19.71, *p* < .001, η_p_^2^ = .46, but not for errors, *F*(1, 23) = 0.01, *p* = .9, η_p_^2^ = .00. Symmetry detection (0.93 s, 6% errors) was faster but not more accurate than repetition detection (1.09 s, 6%).

Line separation was significant for both RT, *F*(2, 46) = 135.04, *p* < .001, η_p_^2^ = .85, and errors, *F*(2, 46) = 29.19, *p* < .001, η_p_^2^ = .56. Post hoc Newman-Keuls analyses (*p* < .05) revealed that regularity detection was both faster and more accurate with small separations (0.89 s, 3% errors) than with medium separations (1.01 s, 6%) and, in turn, that detection was both faster and more accurate with medium separations compared to large separations (1.13 s, 9%).

**Figure 5:**
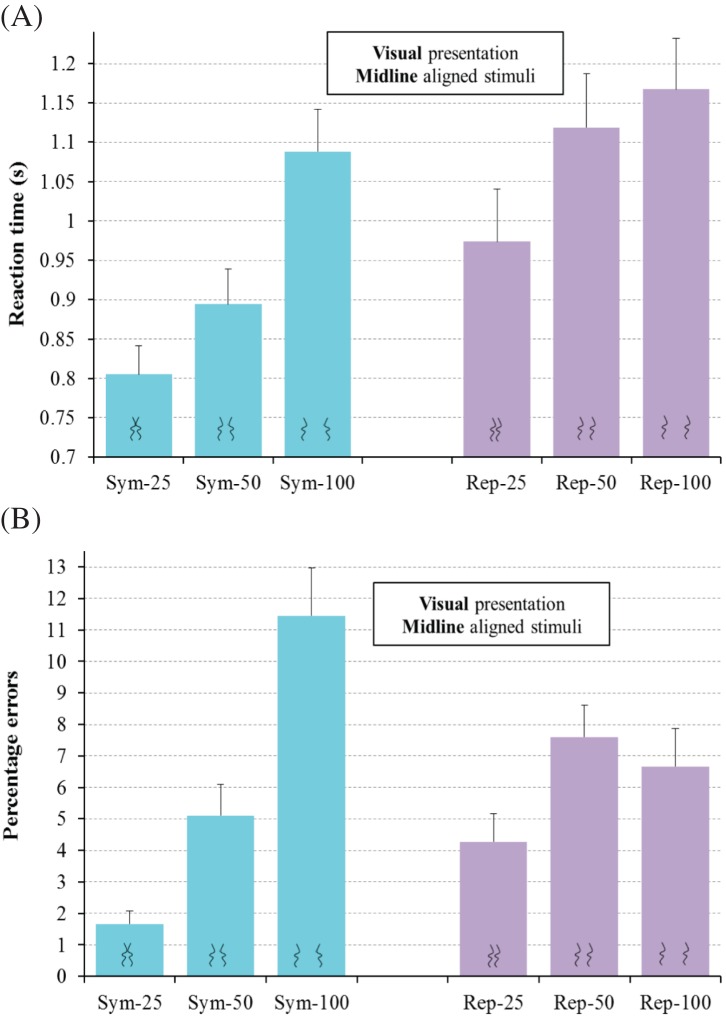
Results for regular trials for Experiment 1 for the visual detection of symmetry (Sym) and repetition (Rep) for line separations of 25 mm, 50 mm, and 100 mm for RT (A) and errors (B). Error bars represent one standard error of the mean. In this, and the remaining figures showing experimental results, the icons at the base of each bar schematically represent the type of stimuli in that condition: symmetrical or repeated with small, medium, or large separations between each pair of lines.

Finally, the interaction of Regularity-type × Line separation was significant for both RT, *F*(2, 46) = 12.32, *p* < .001, η_p_^2^ = .35, and errors, *F*(2, 46) = 11.59, *p* < .001, η_p_^2^ = .34, see Figure 5. To understand this interaction, we calculated the difference between regularity detection for the largest (100 mm) compared to the smallest (25 mm) line separation and conducted an ANOVA on these differences. This revealed that increased line separation (100 mm–25 mm) was significantly more disruptive for detecting symmetry (0.28 s, 10% errors) than for detecting repetition (0.19 s, 2%) for both RT, *F*(1, 23) = 6.74, *p* = .016, η_p_^2^ = .28, and errors, *F*(1, 23) = 12.62, *p* = .002, η_p_^2^ = .35.

### Discussion

In Experiment 1, our hypothesis was that line separation and regularity-type are both factors which provide evidence about objectness. We therefore predicted that effects of line separation should interact with those of regularity-type, with the disruptive effect of increased line separation being greater for symmetry detection than for repetition detection. Our results for visual regularity detection confirmed this prediction. Converging evidence for this interaction between the effects of objectness and regularity-type on vision has been reported when objectness is manipulated using planar shapes like those shown in Figure 1 (Baylis & Driver, 1995; Bertamini, 2010; Bertamini et al., 1997; Koning & Wagemans, 2009; Cecchetto & Lawson, 2016). Further discussion of these results for vision is deferred until we have described the Results of Experiments 2 and 3, which investigated the interaction between regularity-type and line separation for haptic regularity detection.

## Experiment 2

Experiment 2 largely replicated Experiment 1, except that the stimuli were presented haptically, as 3D raised lines, rather than visually, as 2D digital images. We again investigated the effects of type of regularity (symmetry or repetition) and line separation on regularity detection. Based on the results of Experiment 1, we might expect that symmetry should be easier to detect with small line separations, since nearby lines and symmetry may provide consistent evidence that a single object is present, whereas large line separations and symmetry provide conflicting cues about objectness. The opposite pattern might be expected for repetition, with repetition being easier to detect at large line separations, since repetition and distant lines may provide consistent cues that two objects are present. Note, though, that there are independent reasons why regularities might become harder to detect at large line separations (irrespective of whether symmetry or repetition is being detected). For example, participants probably find it harder to align their fingers precisely in space when they are further apart. From debriefing and informal observation, we believe that finger alignment is critical for haptic regularity detection. Any such independent effects would counter the expected advantage for large line separations for repetition (while enhancing the cost for large line separations for symmetry). We therefore simply predicted that, if haptic regularity detection behaves like visual regularity detection, then increased line separation should disrupt symmetry detection more than repetition detection.

However, importantly, when we manipulated perceived objectness in previous experiments using closed-contour, planar stimuli rather than line separation (Cecchetto & Lawson, 2016; see Figure 2), we obtained an interaction between objectness and regularity-type for vision but not for haptics. Based on these findings, if haptics again behaves differently to vision, in Experiment 2 compared to Experiment 1, we would not predict a greater influence of line separation when haptically detecting symmetry compared to repetition.

### Method

#### Participants

The same 24 participants from Experiment 1 took part in Experiment 2, in a second, separate session. This haptic session was always conducted before visual testing occurred in Experiment 1 (on average, 6 days earlier, range 0–15 days). However, for ease of explanation, we described the visual experiment first.

#### Materials

There were 240 stimuli, comprising half of the 480 pairs of lines used in Experiment 1. The pairs of lines were based on 20 of the 40 unique lines used in Experiment 1. These 20 lines were selected to span the range of difficulty that we observed in Experiment 1 of Cecchetto and Lawson (2016), which used the same lines, see Figure 3. This was done by ordering performance for regularity detection for each line from best to worst and then selecting alternate lines.

We used a laser cutter to produce the 3 mm wide × 100 mm tall plastic lines from 5 mm thick acrylic sheets. Pairs of lines were glued onto 15 cm wide × 10 cm tall cardboard bases with the top and bottom of each line aligned with the top and bottom of the base, respectively, so the long axes of each line lay parallel to each other. The dimensions of the stimuli were matched to the dimensions of the stimuli used in Experiment 1 so the top and bottom of each line was positioned 12.5 mm each side of the midpoint of the base for the 25 mm separated lines, 25 mm each side of it for the 50 mm separated lines, and 50 mm each side of it for the 100 mm separated lines.

### Line Separation Discrimination Check

We conducted a rating study to check that participants in Experiment 2 could haptically discriminate between the line separations presented. Twenty-four students from the University of Liverpool (17 females, mean age = 19 years, *SD* = 1.5, range 18–25) volunteered to take part. Twelve participants were allocated to the two-handed exploration group. They felt two lines simultaneously with their two index fingers. The remaining 12 participants were allocated to the one-handed exploration group. They used their right index finger to feel the right line and their right thumb to feel the left line. This rating study used the same procedure and a subset of the trials used in Experiment 2. Each participant completed the same nine trials which were presented in a fixed, pseudorandom order. These comprised three symmetry trials, three repetition trials, and three irregular trials, each with a small, medium, and large line separation. Participants responded verbally as to, first, whether each pair of lines was symmetrical, repeated, or irregular, and then whether each pair of lines was separated by a small, medium, or large gap. Thus, both tasks involved distinguishing between three categories. Accuracy was similar for the regularity discrimination task (21% errors with one hand, 27% with two hands) and the line separation discrimination task (23% errors with one hand, 21% with two hands).

#### Design

This was identical to Experiment 1 except for the following points. Participants did the same block order (symmetry detection then repetition detection or vice versa) as they had done in Experiment 1. However, because regularity detection is much faster for vision than for haptics, and because there were only half the stimuli in Experiment 2 as in Experiment 1, participants only did a quarter of the number of trials in Experiment 2 as in Experiment 1. Participants thus completed 120 of the possible 240 experimental trials in 2 blocks of 60 trials. The 240 trials (20 unique lines × Regular/irregular stimuli × Small/medium/large line separations) were divided into 4 blocks of 60 trials. Each of these blocks included 20 stimuli at each of the three line separations and they also all included three stimuli based on each of the 20 unique lines with half the stimuli being regular and half irregular in each block. Trials within a block were presented in a fixed, pseudorandom order. The assignment of participants to blocks was counterbalanced by dividing the participants into six subgroups of four participants and then, within each subgroup, all four blocks were completed once as the first block and once as the second block.

#### Procedure

**Figure 6:**
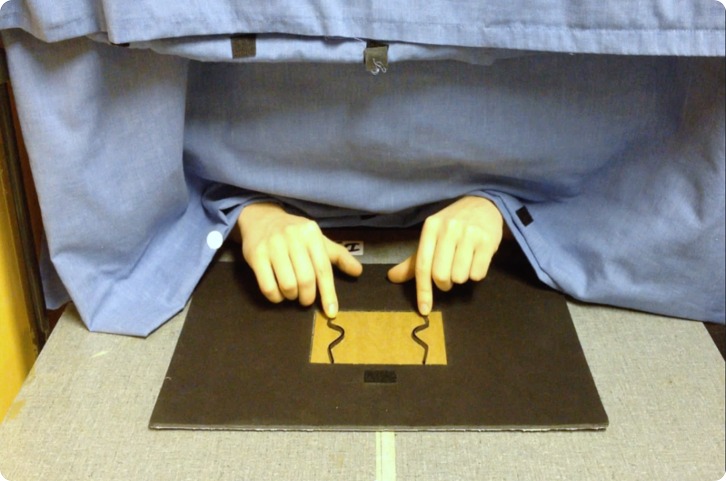
Haptic exploration of a large line separation (100 mm), symmetrical stimulus in Experiment 2 as seen from the experimenter’s perspective. Note the startpoint patch at the top of the stimulus.

This was identical to Experiment 1 except for the following points. Stimuli were presented in front of participants on a 70 cm high table, see Figure 6. A curtain hung directly in front of the participant, around 15 cm inside the edge of the table. Participants put their hands under the curtain, hiding both the stimuli and their hands from view. On the table in front of the curtain there were two labels, “same” on the left and “different” on the right, to remind participants which foot pedal they should use to respond on regular and irregular trials respectively. Participants were instructed to center their body midline with the midpoint of the two response labels and the midpoint of the two foot pedals.

The experimenter placed stimuli, one at a time, in a recess (15 cm wide × 10 cm tall) within a 45 cm wide × 30 cm tall foamboard frame. The frame ensured that the stimuli were presented at a fixed position and orientation. The center of the recess was in line with the participant’s body midline and was 25 cm from the edge of the table and approximately 40 cm from the participant. There was a soft patch on the frame, positioned above the middle of the top of the recess, see Figure 6. Participants rested both of their index fingers on this startpoint patch before beginning each trial, so they started exploring lines from the top.

Before starting the experiment, participants were shown visually four practice stimuli. These stimuli were similar to the experimental stimuli: two were regular, two were irregular, and all had a medium separation. Participants then did four practice trials haptically using these stimuli. They were instructed to feel one line with each of their two index fingers, and to decide whether the lines were regular.

At the start of each trial the experimenter placed a stimulus in the recess, then triggered an audible “go now” signal using the computer. This indicated to the participant that they should move their fingers from the resting position on the startpoint patch, to begin to explore the two lines. Participants were told to respond as quickly and as accurately as possible by pressing a foot pedal. Reaction times (RT) were measured from the offset of the go signal to the participant’s pedal response. Following their response, a high or a low pitch feedback sound was emitted to indicate a correct or a wrong answer respectively.

After the first block of 60 experimental trials, participants were instructed about the new regularity that they would have to detect. They were again shown visually four new practice stimuli which were then used in four haptic practice trials before they did the second block of 60 experimental trials. The experiment took around one hour to complete. Afterwards the experimenter checked to ensure that the participant had not seen any of the stimuli.

### Results

No participant was replaced. Correct RT faster than 1 s or slower than 35 s were removed as outliers (less than 1% of trials).^2^ As in Experiment 1, ANOVAs were conducted on the mean correct RT and on the percentage of errors for regular trials only. Analyses of measures of sensitivity (d′) and bias (c′) are given in the Appendix. There were two within-participants factors in the ANOVAs: regularity-type (symmetry or repetition) and line separation (small, medium, or large).

Regularity-type was significant for both RT, *F*(1, 23) = 6.86, *p* = .015, η_p_^2^ = .23, and errors, *F*(1, 23) = 11.77, *p* = .002, η_p_^2^ = .34. Symmetry detection (7.2 s, 8% errors) was both faster and more accurate than repetition detection (8.7 s, 16%).

Line separation was significant for RT, *F*(2, 46) = 3.80, *p* = .03, η_p_^2^ = .14, and was marginally significant for errors, *F*(2, 46) = 2.97, *p* = .06, η_p_^2^ = .11. The overall pattern was for regularity to be easiest to detect at small separations (7.6 s, 9% errors), in between for medium separations (8.0 s, 12.5%), and hardest for large separations (8.2 s, 15%). However, in post hoc Newman-Keuls analyses only the difference in speed between small and large separations was significant (*p* < .05).

**Figure 7:**
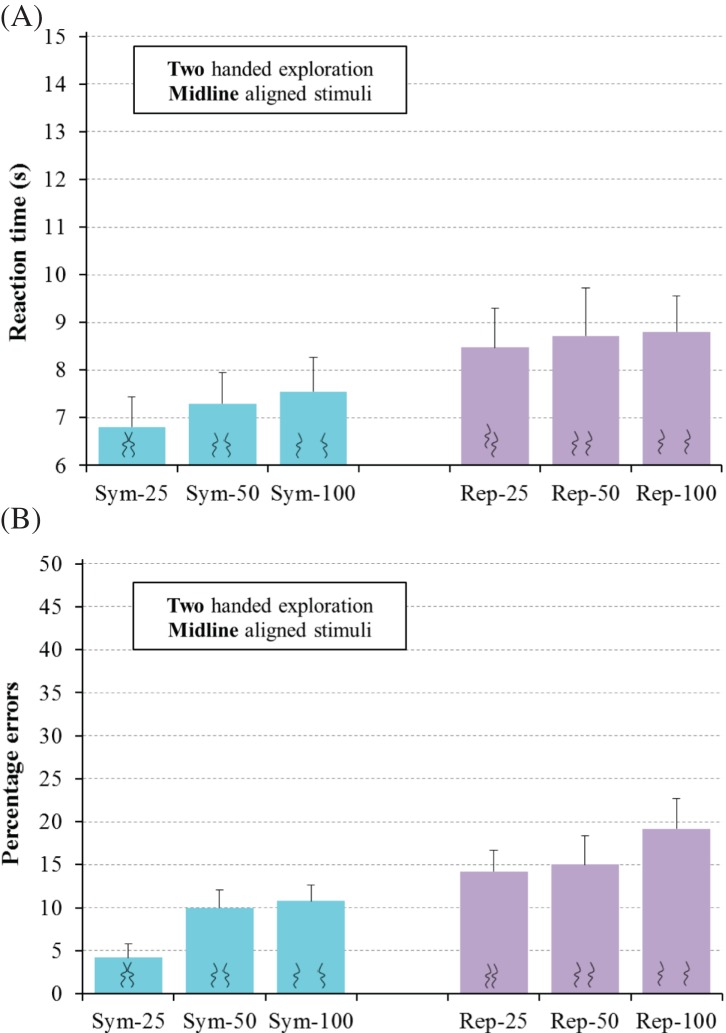
Results for regular trials for Experiment 2 for the haptic detection of symmetry (Sym) and repetition (Rep) for line separations of 25 mm, 50 mm, and 100 mm for RT (A) and errors (B). Error bars represent one standard error of the mean.

Finally, the interaction of Regularity-type × Line separation was not significant for RT, *F*(2, 46) = 0.50, *p* = .6, η_p_^2^ = .02, or for errors, *F*(2, 46) = 0.70, *p* = .5, η_p_^2^ = .03. The effect of line separation was similar for symmetry detection and repetition detection, see Figure 7.

### Discussion

Experiment 2 revealed a modest cost of increasing line separation on haptic regularity detection together with an overall advantage for detecting symmetry compared to repetition. Unlike visual regularity detection in Experiment 1, we did not find a greater cost of line separation when detecting symmetry compared to repetition, for either the RT or the error analyses of regular trials. This difference between the results of Experiments 1 and 2 suggests that modality-specific processing influences the detection of regularities. This conclusion is consistent with the findings of Cecchetto and Lawson (2016) where we compared regularity detection in vision and touch for planar, closed-contour shapes (see Figure 2). However, we should note that the results of the sensitivity analysis revealed an interaction in the predicted direction between the effects of line separation and regularity-type, see the Appendix, so there was some inconsistency in the results. Experiment 3 was therefore conducted to investigate this issue further.

## Experiment 3

Experiment 3 was conducted to probe whether effects on regularity detection reflect perceptual processes unique to haptics rather than reflecting properties of the physical stimuli. This question was addressed by, first, changing the orientation of the lines relative to a body-centered spatial frame of reference and, second, altering the manner of haptic exploration. Both of these manipulations were expected to change modality-specific aspects of perceptual processing while leaving unaltered the stimuli and their surrounding environment. If an understanding of regularity detection tells us about the information available to us in the world then neither manipulation should affect performance. However, if regularity detection is sensitive to how information is acquired and processed then both manipulations may influence performance. The results for separate pairs of lines in Experiments 1 and 2 here, and for planar, closed-contour shapes in Cecchetto and Lawson (2016), suggest that effects of regularity-type and objectness differ for visual versus haptic regularity detection. This supports the latter prediction.

Experiment 3 replicated Experiment 2 except for two main points. First, the stimuli were rotated by 90° so that the axis of regularity ran perpendicular to the body midline (in the across condition) rather than being aligned with it (as in Experiments 1 and 2). When stimuli are aligned with their body midline, participants can represent symmetrical stimuli using a highly salient spatial frame of reference based on the symmetry of their own body (e.g., Ballesteros, Millar, & Reales, 1998). Having the axis of regularity of symmetrical stimuli aligned with the axis of bilateral symmetry of the participant’s own body midline may thus aid symmetry detection relative to repetition detection. However, any benefit from using this salient reference frame should be weaker, or absent, if the axis of regularity of symmetrical stimuli is perpendicular to the axis of bilateral symmetry of the participant’s own body midline, as it was for across stimuli in Experiment 3. We therefore predicted that symmetry detection would be harder in the across condition in Experiment 3 than in the aligned condition in Experiment 2.

**Figure 8:**
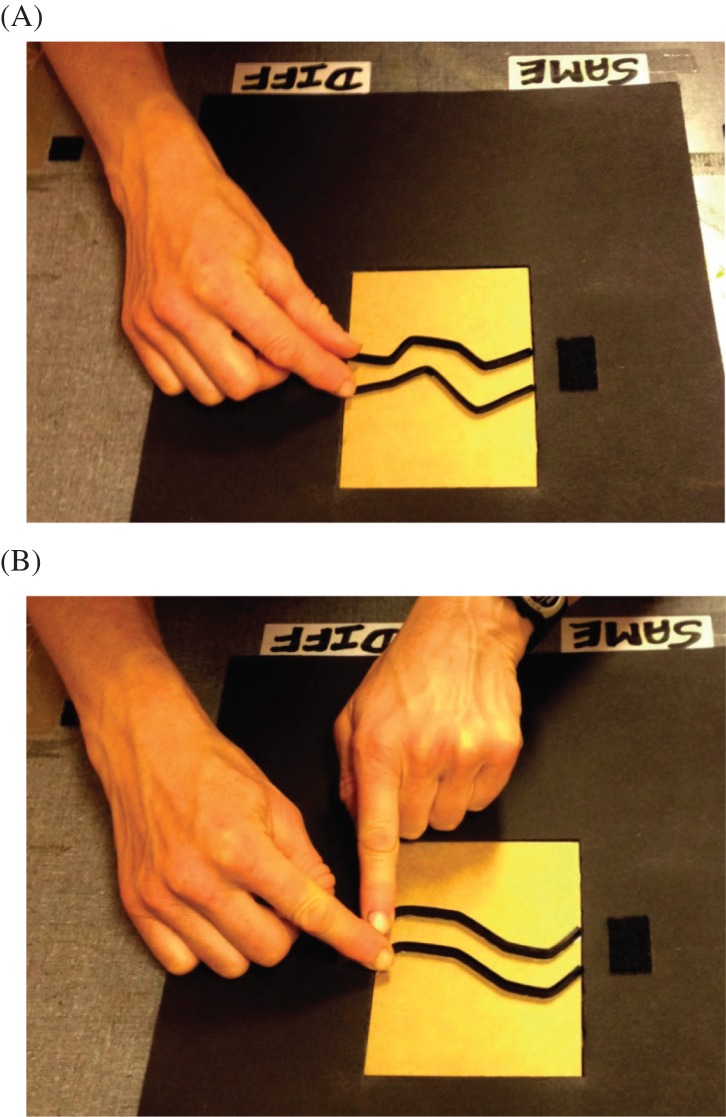
A participant in Experiment 3 shown (A) using one hand to explore a pair of irregular lines separated by 25 mm and, (B) using two hands to explore a pair of repeated lines separated by 25 mm. Note that both stimuli are oriented such that the axis of regularity is perpendicular to the participant’s body midline. This contrasts to Experiments 1 and 2 where the stimuli were aligned with the body midline (see Figures 4 and 6). For the purpose of these photographs, the curtain was raised to show the response labels which reminded the participant which foot pedal to use. The black startpoint patch is shown to the right of the stimulus (so it was on the left side for the participant).

Second, in Experiment 3 the manner of stimulus exploration was manipulated between participants. One group used the same, two-handed exploration tested in Experiment 2, with their two index fingers each feeling one of the two lines, see Figure 8. A second group explored stimuli using only one hand. They used their right index finger to explore the top line and their right thumb to explore the bottom line. We reasoned that, in addition to regularity-type and line separation, the manner of exploration could provide a third, independent, and modality-specific cue to objectness. In our everyday interactions we often explore and hold a single object in one of our hands. Thus, if we feel two lines with two parts of one hand (here, the thumb and index finger) this may be used as a cue that we are feeling two parts of the same object rather than feeling two different objects. In contrast, we frequently touch and use two different objects with our right and our left hands. Thus, if each of our index fingers feels a different line, this may be used as a cue that we are feeling two different objects. One-object interpretations of line pairs may therefore be more consistent with one-handed exploration than two-handed exploration and vice versa for two-objects interpretations. If so, then symmetry should be easier to detect for one-handed exploration (since here cues from both regularity-type and exploration would consistently indicate that one object was present) compared to two-handed exploration (where cues about objectness would be conflicting) and vice versa for repetition (which should be easier to detect with two-handed than one-handed exploration).

### Method

The design, stimuli, and procedure in Experiment 3 were identical to Experiment 2 except for the following points. Thirty-two students from the University of Liverpool (22 females, mean age = 21 years, *SD* = 3.8, range 18–31) volunteered to take part in the experiment. All of the stimuli were rotated 90° counterclockwise so that the orientation of the regularity was perpendicular to the participant’s body midline. The frame that the stimuli were placed in was also rotated 90° counterclockwise so the startpoint patch on which participants rested their fingers at the beginning of each trial was on the left side of the frame rather than on the top of the frame, see Figure 8. Participants started each trial by moving their fingers from left to right rather than from top to bottom. Half of the participants were instructed to explore the two lines simultaneously with their two index fingers, as in Experiment 2. The remaining participants were instructed to explore the two lines using their right hand only, with their right index finger feeling the top line and their right thumb feeling the bottom line, see Figure 8.

### Results

No participant was replaced. There was one empty cell in the RT data for one participant in the one-handed exploration group, which was filled by the mean for that condition. As in Experiment 2, correct RT faster than 1 s or slower than 35 s were removed as outliers (less than 1% of trials), and ANOVAs were conducted on the mean correct RT and on the percentage of errors for regular trials only.^2^ For clarity of presentation, we give the results for the two exploration groups separately below. However, ANOVAs comparing the two groups tested in Experiment 3, and ANOVAs comparing the results of Experiment 2 to the two-handed exploration group in Experiment 3, are given in the Appendix. Analyses of measures of sensitivity (d′) and bias (c′) for each group are also given in the Appendix.

#### One-Handed Exploration Group

Participants used the thumb and index finger of their right hand to explore stimuli that were oriented to be perpendicular to their body midline. There were two within-participants factors: regularity-type (symmetry or repetition) and line separation (small, medium, or large).

Regularity-type was significant for both RT, *F*(1, 15) = 6.32, *p* = .02, η_p_^2^ = .30, and errors, *F*(1, 15) = 5.89, *p* = .03, η_p_^2^ = .28. We found the advantage for symmetry detection (8.2 s, 14% errors) over repetition detection (9.7 s, 20%) that we obtained in Experiment 2.

Line separation was significant for both RT, *F*(2, 30) = 30.54, *p* < .001, η_p_^2^ = .67, and errors, *F*(2, 30) = 13.54, *p* < .001, η_p_^2^ = .47. Post hoc Newman-Keuls analyses (*p* < .05) revealed that regularity detection was harder for large separations (10.2 s, 29% errors) than for small (8.0 s, 7.5%) and medium (8.6 s, 14%) separations, with no significant difference between small and medium separations.

**Figure 9:**
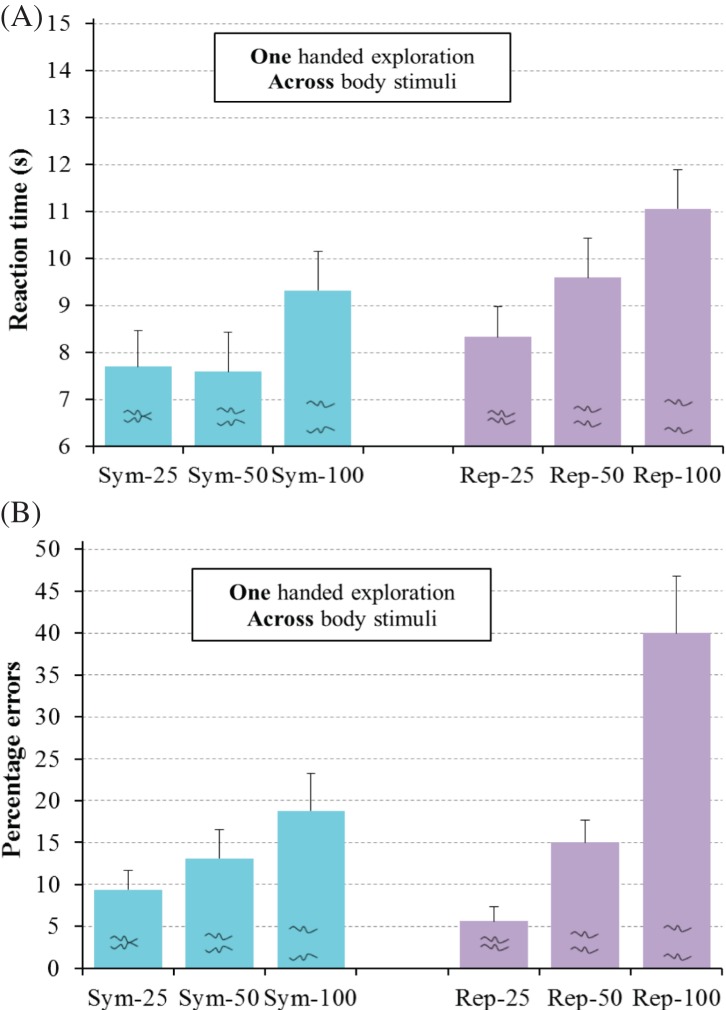
Results for regular trials for the one-handed exploration group in Experiment 3 for the haptic detection of symmetry (Sym) and repetition (Rep) for line separations of 25 mm, 50 mm, and 100 mm for RT (A) and errors (B). Error bars represent one standard error of the mean.

The interaction of Regularity-type × Line separation was not significant for RT, *F*(2, 30) = 1.30, *p* = .3, η_p_^2^ = .08, but it was for errors, *F*(2, 30) = 8.52, *p* = .001, η_p_^2^ = .36, see Figure 9. To understand this interaction we calculated the difference between regularity detection for the largest (100 mm) compared to the smallest (25 mm) line separation and conducted an ANOVA on these differences. This revealed that the cost of increased line separation (100 mm–25 mm) on accuracy was significantly less for symmetry (9% errors) than for repetition (34%), *F*(1, 15) = 15.96, *p* = .001, η_p_^2^ = .51. Note that this pattern shows the reverse interaction to that which we found for vision in Experiment 1 where the accuracy of repetition detection was more sensitive to line separation than was symmetry detection.

#### Two-Handed Exploration Group

Participants used both of their index fingers to explore stimuli that were oriented to be perpendicular to their body midline. There were again two within-participants factors: regularity-type (symmetry or repetition) and line separation (small, medium, or large).

Regularity-type was significant for both RT, *F*(1, 15) = 12.43, *p* = .003, η_p_^2^ = .45, and errors, *F*(1, 15) = 37.27, *p* < .001, η_p_^2^ = .71. Symmetry detection (13.2 s, 28% errors) was both slower and less accurate than repetition detection (10.7 s, 9%). Note that this clear-cut advantage for detecting repetition contrasts to both the symmetry advantage found here in Experiment 3 for one-handed haptic exploration of across-body stimuli, and the symmetry advantage found in Experiment 2, for two-handed haptic regularity detection of body midline-aligned stimuli, as well as the symmetry advantage found in Experiment 1, for visual regularity detection of body midline-aligned stimuli.

Line separation was significant for RT, *F*(2, 30) = 5.38, *p* = .01, η_p_^2^= .26, but not for errors, *F*(2, 30) = 0.43, *p* = .6, η_p_^2^ = .03. Post hoc Newman-Keuls analyses (*p* < .05) revealed that regularity detection was slower for large separations (12.6 s, 20% errors) than for medium (11.4 s, 18%) and small (11.7 s, 18%) separations, with no significant difference between medium and small separations.

**Figure 10:**
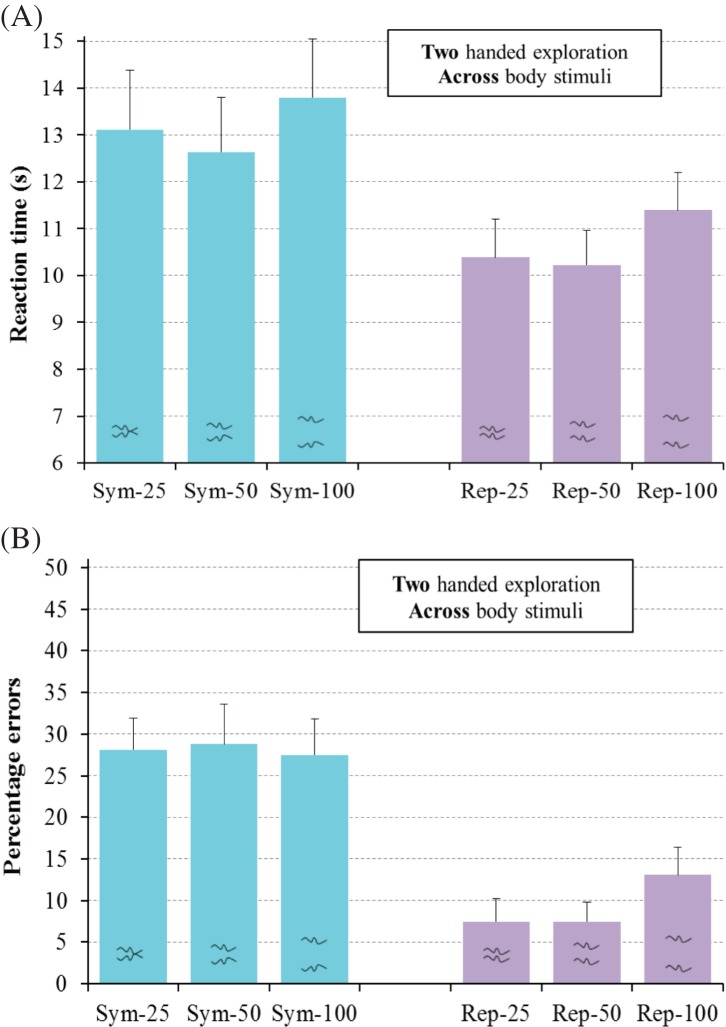
Results for regular trials for the two-handed exploration group in Experiment 3 for the haptic detection of symmetry (Sym) and repetition (Rep) for line separations of 25 mm, 50 mm, and 100 mm for RT (A) and errors (B). Error bars represent one standard error of the mean.

The interaction of Regularity-type × Line separation was not significant for RT, *F*(2, 30) = 0.17, *p* = .8, η_p_^2^ = .01, or for errors, *F*(2, 30) = 1.18, *p* = .3, η_p_^2^ = .07, see Figure 10, with a similar slowing at larger separations for symmetry and repetition detection.

### Discussion

The main findings from Experiment 3 involved interactions between regularity-type and three other factors: line separation, exploration type, and stimulus orientation. We discuss each of these in turn. First, these results confirmed the difference between haptic and visual regularity detection which we observed when comparing the results of Experiments 1 and 2. For the two-handed exploration group, effects of line separation were similar for symmetry detection and for repetition detection, replicating the results for two-handed haptic exploration in Experiment 2, see Figure 10. For the one-handed exploration group, line separation influenced the accuracy of repetition detection more than that of symmetry detection, see Figure 9. This interaction was the reverse of the interaction which we observed for visual regularity detection, in Experiment 1, where increased line separation disrupted the detection of symmetry more than repetition. For vision, in Experiment 1, the results supported the hypothesis that small line separations and symmetry provide consistent evidence for the presence of a single object while large line separations and repetition provide consistent evidence for the presence of multiple objects. However, this account was not supported by the results for haptics, in Experiment 2 or 3.

Second, regularity detection was strongly influenced by whether one or two hands were used to explore across stimuli, where the axis of regularity ran perpendicular to the body midline. Symmetry was easier to detect than repetition for one-handed exploration whereas repetition was easier to detect than symmetry for two-handed exploration. These results were consistent with our predictions based on the hypothesis that both symmetry and one-handed exploration are cues for the presence of one object, whereas both repetition and two-handed exploration are cues for the presence of multiple objects.

Third, considering only two-handed exploration, in Experiment 2 symmetry was easier to detect than repetition when the axis of regularity of the stimuli was aligned with the body midline. Thus, here we found the usual symmetry advantage. In contrast, for the two-handed group in Experiment 3, repetition was easier to detect than symmetry when the axis of regularity ran across the body midline. This result suggests that, in Experiment 2 only, an egocentric, body-centered (rather than an allocentric, world-centered) spatial frame of reference could be used to represent stimuli aligned with the body midline. Here symmetry detection was privileged relative to repetition detection because the axis of symmetry of the stimuli was coincident with a reference frame based on the axis of bilateral symmetry of the participant’s own body. Thus, the orientation of stimuli relative to the body midline appears to play an important role in haptic symmetry detection.

We found an advantage for haptically detecting symmetry relative to repetition for both two-handed exploration of midline-aligned stimuli in Experiment 2, and for one-handed exploration of across-body stimuli in Experiment 3. Thus, an advantage for haptic detection of repetition occurred only when *both* the manner of exploration was consistent with a two-objects interpretation of the stimulus (i.e., two-handed exploration, favoring repetition detection) *and* when participants could not easily take advantage of body-centered spatial frames of reference (for across stimuli, where the axis of symmetry of stimuli was perpendicular to the axis of bilateral symmetry of the participant’s own body). Thus, in general, symmetry appears to be easier to detect than repetition for haptics, consistent with what has long been established for vision (Julesz, 1971).

## General Discussion

The present studies investigated two issues. First, we used truly repeated rather than anti-repetition stimuli to seek evidence for the claim that there is a one-object advantage for detecting symmetry and a two-objects advantage for detecting repetition (Koning & Wagemans, 2009; Van der Helm & Treder, 2009). Second, we investigated whether effects found for regularity detection reflect internal, modality-specific processing or if they reveal differences arising directly from the presence of regularities out in the external, physical world. The overall motivation for examining these issues was to gain insights into what it means to be an object in vision and in touch. We manipulated three different potential cues to objectness: the type of regularity being detected, the separation between task-critical lines, and whether one hand versus two hands felt stimuli in haptic tasks. We hypothesized that symmetry, small line separations, and one-handed exploration would all provide evidence that a single object was present, whereas repetition, large line separations, and two-handed exploration would all provide evidence that two objects were present. Our results revealed that regularity detection is strongly influenced by all three possible cues to objectness, and that these effects are modulated by the modality of stimulus presentation, and, for haptics, by the ease of use of egocentric, body-centered spatial reference frames. We found the predicted interaction of objectness by regularity-type in some, but not all, cases and this depended on whether stimuli were presented visually or haptically, as detailed below. We argue that these effects on regularity detection may mainly inform us about modality-specific encoding and processing strategies used by vision and touch and, thus, that they may not reflect intrinsic properties of objects in the physical world. Three of our results support this claim.

First, we compared line separation effects for haptic and visual regularity detection. In the context of visual perception, it has often been claimed that symmetry is used as a cue for the presence of a single, bilaterally symmetric object, while repetition is used as a cue for the presence of multiple, similarly shaped objects (Koning & Wagemans, 2009; Van der Helm & Treder, 2009). These claims are plausible but, as reviewed in the Introduction, there is surprisingly little evidence for them. For line separation we investigated a novel prediction that during regularity detection our perceptual processes may take advantage of the fact that pairs of nearby lines are more likely to belong to a single object whereas pairs of more distant lines are more likely to belong to two different objects.

For vision, in Experiment 1, we found the predicted interaction between regularity-type (symmetry vs. repetition) and the distance between lines. For vision, in Experiment 1, the cost of increased line separation was greater for symmetry than for repetition. This was as predicted since nearby, symmetrical lines provide consistent cues that a single object is present, whereas these cues are in conflict for well-separated, symmetrical lines. The opposite predictions were made for repetition, with well-separated, repeated lines providing consistent cues that multiple, similar objects are present, whereas these cues are in conflict for nearby, repeated lines. The results of Experiment 1 are consistent with our previous findings using symmetrical and anti-repetition planar shapes (see Figure 2) where visual symmetry was easier to detect for one-object (as opposed to two-objects) stimuli and the reverse was true for repetition (Cecchetto & Lawson, 2016).

In contrast, for haptics, in Experiments 2 and 3, there was an overall advantage for detecting regularities across pairs of nearby (as opposed to well-separated) lines, but no reliable interaction between regularity-type and line separation. Instead, the cost of increased line separation was similar for symmetry and for repetition detection, except for the one-handed group in Experiment 3. In this latter case, the opposite interaction was found to that observed for vision, namely a greater advantage for nearby lines for detecting repetition than for symmetry.^3^ Again, these results are similar to our previous findings using planar shapes (Cecchetto & Lawson, 2016), where regularities were easier to detect within a single object (as opposed to across two objects) for both symmetry and repetition in haptics.

Thus, in both the present studies and in Cecchetto and Lawson (2016), we reliably found the predicted interaction between objectness and regularity-type for vision, but not for haptics. For vision, but not for haptics, these results are consistent with small line separations and symmetry providing consistent cues that a single object is present while large line separations and repetition provide consistent cues that two objects are present. We do not, as yet, have a good account of why vision and touch behave differently in this case. To address this issue, we have conducted further studies in which we have manipulated the time course of presentation of stimuli to vision and whether stimuli are presented all at once, or are viewed through a moving aperture (Cecchetto & Lawson, 2015).

Second, we tried to manipulate perceived objectness by changing how participants haptically explored the stimuli. We reasoned that pairs of lines explored with one hand are more likely to be interpreted as belonging to a single object, whereas pairs of lines explored with two separate hands may be more likely to be interpreted as belonging to two different objects.

To test this hypothesis, in Experiment 3 we compared haptic regularity detection of across stimuli using the index fingers of both hands, versus using the thumb and index finger of the right hand. Averaging over the effects of line separation, symmetry was easier to detect than repetition for one-handed exploration whereas the reverse was true for two-handed exploration. This result is consistent with one-handed exploration and symmetry providing consistent cues that a single object is present while two-handed exploration and repetition provide consistent cues that two objects are present, making regularity detection easier overall in both cases. In contrast, regularity detection was harder overall when cues provided conflicting information about the number of objects present (for one-handed exploration of repetition, and for two-handed exploration of symmetry).

Regularity detection is probably also influenced by other aspects of haptic exploration which were not manipulated experimentally in the present studies. From pilot testing, and informal observation, it appears that regularity detection depends critically on aligning in time the inputs from exploring two, matched parts of a regular stimulus. In addition, the position of a finger on a contour or line (on the left or right side or on top) may influence how the shape of that edge is perceived. Future research should test how such changes in exploration strategies may influence the detection of regularities and the perception of objectness.

Third, we compared two-handed haptic regularity detection for stimuli aligned to the body midline of the participant (Experiment 2) and for stimuli rotated so that the axis of regularity ran perpendicular to the body midline (Experiment 3). Symmetry was easier to detect than repetition for stimuli aligned with the body midline. Here, the body’s own axis of bilateral symmetry provided a salient spatial frame of reference which was aligned with the axis of regularity of symmetrical stimuli. In contrast, repetition was easier to detect than symmetry when there was no privileged reference frame for symmetry detection because- the axis of regularity ran across the body midline.

Across all three of these comparisons, the same symmetrical and repeated stimuli were presented at the same line separations. Only the manner of processing differed across conditions (modality: vision vs. haptics; manner of haptic exploration: one-handed vs. two-handed; and orientation relative to the body midline: aligned vs. across). Although the physical stimuli presented were not altered by these three manipulations, each had a clear effect on regularity detection, indicating the powerful influence of differences in perceptual encoding and processing.

Finally, we should highlight the fact that although in our studies we propose that we have manipulated several potential cues to objectness, even in vision, it has proven difficult to provide a formal definition of objectness (Feldman, 2003), while in haptics this topic does not appear to have been addressed at all. We do not claim that we have objectively varied objectness nor do we consider that objectness is a clear-cut, all-or-nothing attribute of stimuli. Previous studies which investigated the interaction between regularity detection and objectness using anti-repetition (Baylis & Driver, 1995; Bertamini, 2010; Bertamini et al., 1997; Cecchetto & Lawson, 2016; Koning & Wagemans, 2009) used a mixture of cues to define objectness including closure, regularities, color, luminance, 3D projections, and stratification in depth to distinguish one-object from two-objects stimuli. Consistent with this approach, we suggest that multiple cues to objectness are extracted from perceptual inputs. Our results show that, in addition to those cues listed above, line separation and manner of exploration may play a significant role in specifying objectness. An important issue for future research will be to try to understand the relative importance of these cues in determining objectness, how they are combined and how any conflicts between them are resolved. In particular, the manipulations used in the present studies provide a promising means of investigating how objectness is specified for our sense of touch.

In conclusion, we found several interactions consistent with the predictions of an account that proposes that regularity-type, line separation, and manner of exploration can all influence regularity detection because they are all informative about the nature of objectness. In contrast to the results for vision, the results for the interaction of regularity-type by line separation for haptics did not support this account. It is not clear why we did not obtain the latter interaction, but this result seems reliable, given that we obtained a similar result when objectness was manipulated more directly, using planar shapes and anti-repetition (Cecchetto & Lawson, 2016, see Figure 2), rather than line separation and true repetition as used here. Together, these results inform us about, first, what cues may be used to determine objectness (regularity-type and line separation for vision; regularity-type and manner of exploration for haptics) and, second, what we can learn from effects on regularity detection. First, these results support the proposal that symmetry, small line separations (for vision but not haptics), and one-handed exploration (for haptics) are all used as cues that a single object is present, while repetition, larger line separations (for vision but not haptics), and two-handed exploration (for haptics) are all used as cues that multiple objects are present. Regularity detection was influenced by all of these potential cues to objectness as well as by the spatial reference frame that could be used to represent the stimuli. Second, these results suggest that effects on regularity detection do not primarily reflect intrinsic, structural properties of physical objects in the world. Several of our manipulations had clear effects on regularity detection despite causing little or no change to physical properties of the stimuli, namely the modality of presentation, the manner of exploration, and the availability of egocentric reference frames. Our findings instead suggest that regularity detection effects may be most informative about modality-specific differences in how stimuli are encoded and processed across vision and touch. This conclusion is consistent with the claims of Feldman (2003) that understanding the nature of objectness will involve specifying how our subjective, internal, perceptual representations are organized, rather than informing us about how the objective, external world is structured.

## Footnotes

^1^ Due to a programming error in Experiment 1, the irregular trials for one of the 40 unique lines incorrectly showed regular stimuli, so the data for these six trials per participant were removed from all analyses.

^2^ Raw data is available to download from Experiment 1 at www.liv.ac.uk/~rlawson/GapPaperExpt1Data.txt, from Experiment 2 at www.liv.ac.uk/~rlawson/GapPaperExpt2Data.txt, and from Experiment 3 at www.liv.ac.uk/~rlawson/GapPaperExpt3Data.txt.

^3^ We speculate that this interaction might reflect the ease of controlling repeated versus symmetrical index finger and thumb movements as the right hand moves across the body during this task. We invite the reader to try this by moving their right hand across the surface of a table to follow imaginary lines.

## Appendix

### Further Analyses

Additional ANOVAs were conducted on measures of sensitivity (d′) and bias (c′) for Experiments 1, 2, and 3 are described below, as well as ANOVAs comparing the two groups tested in Experiment 3, and comparing the results of Experiment 2 to the two-handed exploration group from Experiment 3:

#### Experiment 1: Sensitivity and Bias Analyses

For *sensitivity* (d′), regularity-type was not significant, *F*(1, 23) = 0.30, *p* = .59, η_p_^2^ = .01. Sensitivity was similar for symmetry detection (3.19) and repetition detection (3.14). Line separation was significant, *F*(2, 46) = 25.74, *p* < .001, η_p_^2^ = .53. Post hoc Newman-Keuls analyses (*p* < .05) revealed that sensitivity to regularity detection was greater with small separations (3.43) than with medium separations (3.11) and, in turn, that sensitivity was greater with medium separations compared to large separations (2.95). Finally, the interaction of Regularity-type × Line separation was significant, *F*(2, 46) = 8.49, *p* = .001, η_p_^2^ = .27. To understand this interaction we calculated the difference between the sensitivity of regularity detection for the largest (100 mm) compared to the smallest (25 mm) line separation and conducted an ANOVA on these differences. This revealed that increased line separation (100 mm–25 mm) caused a significantly greater reduction in sensitivity for detecting symmetry (0.76) than for detecting repetition (0.20), *F*(1, 23) = 12.10, *p* = .002, η_p_^2^ = .35.

For *bias* (c′), regularity-type was not significant, *F*(1, 23) = 0.10, *p* = .76, η_p_^2^ = .00. Bias was similar for symmetry detection (−0.09) and repetition detection (−0.08). Line separation was significant, *F*(2, 46) = 13.71, *p* < .001, η_p_^2^ = .37. Post hoc Newman-Keuls analyses (*p* < .05) revealed that bias was greater with small separations (−0.22) than either medium (−0.07) or large (0.02) separations, with no difference between these two. Finally, the interaction of Regularity-type × Line separation was significant, *F*(2, 46) = 6.74, *p* = .003, η_p_^2^ = .23. To understand this interaction we calculated the difference between the sensitivity of regularity detection for the largest (100 mm) compared to the smallest (25 mm) line separation and conducted an ANOVA on these differences. This revealed that increased line separation (100 mm–25 mm) reduced bias more for detecting symmetry (0.38) than for detecting repetition (0.08), *F*(1, 23) = 10.78, *p* = .003, η_p_^2^ = .32. A negative bias indicates a bias to say a regularity was present so this interaction reflected a greater bias to say that symmetry was present than that repetition was present at small line separations.

#### Experiment 2: Sensitivity and Bias Analyses

There were no significant effects for the *bias* analysis. For *sensitivity* (d′), regularity-type was significant, *F*(1, 23) = 10.85, *p* = .003, η_p_^2^ = .42. Sensitivity was greater for symmetry detection (1.92) than repetition detection (1.56). Line separation was significant, *F*(2, 46) = 4.70, *p* = .014, η_p_^2^ = .17. The overall pattern was for sensitivity to regularity detection to be greatest at small separations (1.95), in between for medium separations (1.66), and smallest for large separations (1.63). However, in post hoc Newman-Keuls analyses only the difference in sensitivity between small and large separations was significant (*p* < .05). Finally, the interaction of Regularity-type × Line separation was significant, *F*(2, 46) = 3.94, *p* = .026, η_p_^2^ = .15. This contrasts to the RT and error analyses on regular trials only reported in the main Results section of Experiment 2. To understand this interaction we calculated the difference between the sensitivity of regularity detection for the largest (100 mm) compared to the smallest (25 mm) line separation and conducted an ANOVA on these differences. This revealed a significantly greater reduction in sensitivity at increased line separations (100 mm–25 mm) for detecting symmetry (0.52) rather than repetition (0.17), *F*(1, 23) = 6.55, *p* = .018, η_p_^2^ = .22, as we now mention in the Discussion of Experiment 2.

#### Experiment 3: One-Handed Exploration Group – Sensitivity and Bias Analyses

Participants used the thumb and index finger of their right hand only to explore stimuli that were oriented to be perpendicular to their body midline. There were two within-participants factors: regularity-type (symmetry or repetition) and line separation (small, medium, or large).

For *sensitivity* (d′)*,* regularity-type was significant, *F*(1, 15) = 19.23, *p* = .001, η_p_^2^ = .56, with greater sensitivity for symmetry detection (1.93) than repetition detection (1.33). Line separation was also significant, *F*(2, 30) = 28.044, *p* < .001, η_p_^2^ = .65. Post hoc Newman-Keuls analyses (*p* < .05) revealed that sensitivity to regularity detection was greater at small separations (2.23) than at medium (1.54) separations which, in turn, was greater than at large (1.13) separations (*p* < .05). The interaction of Regularity-type × Line separation was not significant, *F*(2, 30) = 2.27, *p* = .1, η_p_^2^ = .13.

For *bias* (c′)*,* regularity-type was not significant, *F*(1, 15) = 1.33, *p* = .2, η_p_^2^ = .08. Line separation was significant, *F*(2, 30) = 4.38, *p* = .02, η_p_^2^ = .23. Post hoc Newman-Keuls analyses (*p* < .05) revealed that bias was greater at large (−0.06) than at medium (−0.35) separations with no significant differences involving small (−0.24) separations (*p* < .05). The interaction of Regularity-type × Line separation was significant, *F*(2, 30) = 8.75, *p* = .001, η_p_^2^ = .37. Post hoc Newman-Keuls analyses (*p* < .05) revealed that, for symmetry, there were no significant differences between bias at large (−0.19), medium (−0.22), and small (−0.10) separations, whereas for repetition, bias was greater at large (0.06) than at medium (−0.48) and small (−0.38) separations. To understand this interaction we calculated the difference between the sensitivity of regularity detection for the largest (100 mm) compared to the smallest (25 mm) line separation and conducted an ANOVA on these differences. This revealed that increased line separation (100 mm–25 mm) altered bias for detecting symmetry (from −0.10 at 25 mm to −0.19 at 100 mm, a difference of −0.09) in the opposite direction to bias for detecting repetition (from −0.38 at 25 mm to 0.06 at 100 mm, a difference of 0.44), *F*(1, 15) = 21.28, *p* < .001, η_p_^2^ = .59. A negative bias indicates a bias to say a regularity was present.

#### Experiment 3: Two-Handed Exploration Group – Sensitivity and Bias Analyses

Participants used both of their index fingers to explore stimuli that were oriented to be perpendicular to their body midline. There were two within-participants factors: regularity-type (symmetry or repetition) and line separation (small, medium, or large).

For *sensitivity* (d′)*,* regularity-type was significant, *F*(1, 15) = 30.53, *p* < .001, η_p_^2^ = .67. Unlike the one-handed exploration group, sensitivity for the two-handed exploration group was less for symmetry detection (1.02) than for repetition detection (1.65). Line separation was not significant, *F*(2, 30) = 0.45, *p* = .6, η_p_^2^ = .03. Sensitivity was similar at small (1.30), medium (1.41), and large (1.29) separations. The interaction of Regularity-type × Line separation was significant, *F*(2, 30) = 6.06, *p* = .006, η_p_^2^ = .29. To understand this interaction we calculated the difference between the sensitivity of regularity detection for the largest (100 mm) compared to the smallest (25 mm) line separation and conducted an ANOVA on these differences. This revealed that the effect of increased line separation (100 mm–25 mm) was significantly different on symmetry detection and repetition detection, *F*(1, 15) = 8.43, *p* = .01, η_p_^2^ = .36. Increased line separation increased the sensitivity of detecting symmetry (by 0.31), but it reduced the sensitivity of detecting repetition (by 0.32). Thus, in the reverse of the results for visual regularity detection, increased line separation made symmetry detection easier but repetition detection harder.

For *bias* (c′)*,* regularity-type was not significant, *F*(1, 15) = 17.25, *p* = .001, η_p_^2^ = .54, with less bias for symmetry (−0.14) than for repetition (−0.47). Line separation was not significant, *F*(2, 30) = 0.71, *p* = .5, η_p_^2^ = .05. The interaction of Regularity-type × Line separation was significant, *F*(2, 30) = 5.90, *p* = .007, η_p_^2^ = .28. To understand this interaction we calculated the difference between the sensitivity of regularity detection for the largest (100 mm) compared to the smallest (25 mm) line separation and conducted an ANOVA on these differences. This showed no significant difference of an increased line separation (100 mm–25 mm) on the bias for detecting symmetry (0.11) and for detecting repetition (0.06), *F*(1, 15) = 0.11, *p* = .7, η_p_^2^ = .01.

#### Experiment 3: Comparing the One-Handed and Two-Handed Exploration Groups – RT and Error Analyses for Regular Trials and Sensitivity Analyses

There were two within-participants factors: regularity-type (symmetry or repetition) and line separation (small, medium, or large) and one between-participants factor of exploration (one-handed or two-handed). Exploration was significant for RT, *F*(1, 30) = 6.85, *p* = .01, η_p_^2^ = .19, but not for errors, *F*(1, 30) = 0.31, *p* = .5, η_p_^2^ = .01. Overall, one-handed exploration (8.9 s, 17% errors) was faster than two-handed exploration (11.9 s, 19%). In addition, two of the two-way interactions were significant for both RT and errors: for Exploration × Regularity-type, for RT, *F*(1, 30) = 18.68, *p* < .001, η_p_^2^ = .38, for errors, *F*(1, 30) = 38.48, *p* < .001, η_p_^2^ = .56, for Exploration × Line separation, for RT, *F*(2, 60) = 4.22, *p* = .02, η_p_^2^ = .12, for errors, *F*(2, 60) = 7.19, *p* = .002, η_p_^2^ = .19. The third interaction was significant for errors only: Regularity-type × Line separation, for RT, *F*(2, 60) = 1.38, *p* = .2, η_p_^2^ = .04, for errors, *F*(2, 60) = 8.69, *p* < .001, η_p_^2^ = .22. The three-way interaction was not significant for RT and it was marginally significant for errors. The significant interactions arose mainly from the differences in performance of the two exploration groups, see Figures 9 and 10. ANOVAs conducted for each group separately are given in the Results section of Experiment 3.

As in Experiment 1, we also calculated the difference between regularity detection for the largest (100 mm) compared to the smallest (25 mm) line separation and we then repeated the above ANOVA using these (100 mm–25 mm) differences and without the line separation factor. Exploration was significant for both RT, *F*(1, 30) = 5.71, *p* = .02, η_p_^2^ = .16, and errors, *F*(1, 30) = 10.42, *p* = .003, η_p_^2^ = .26. Increased line separation disrupted one-handed regularity detection (cost of 2.2 s on RT and 22% on errors) much more than two-handed regularity detection (0.8 s, 3%). This supports our hypothesis that small line separations and one-handed exploration provide consistent cues that a single object is present while large line separations and two-handed exploration both provide evidence that multiple objects are present. Regularity-type was not significant for RT, *F*(1, 30) = 1.47, *p* = .24, η_p_^2^ = .05, but it was for errors, *F*(1, 30) = 16.53, *p* < .001, η_p_^2^ = .36. Increased line separation disrupted the accuracy of symmetry detection (cost of 1.2 s on RT and 4% on errors) less than that of repetition detection (1.9 s, 20%). This finding does not support the general claim that symmetry provides a cue for the presence of a single object while repetition provides evidence that multiple objects are present. Instead, this finding is consistent with the results of both Experiments 1 and 2 here, and Cecchetto and Lawson (2016), which suggest that there is a one-object advantage for symmetry detection and a two-objects advantage for repetition detection for vision, but not for haptics. Finally, the interaction of Exploration × Regularity-type was not significant for RT, *F*(1, 30) = 0.46, *p* = .50, η_p_^2^ = .02, but it was for errors, *F*(1, 30) = 5.95, *p* = .02, η_p_^2^ = .17. Post hoc Newman-Keuls analyses (*p* < .05) revealed that, for the one-handed exploration group, increased line separation disrupted accuracy less for symmetry (cost of 1.6 s on RT and 9% on errors) than for repetition (2.7 s, 34%) detection. For the two-handed exploration group, there was no significant difference in costs between symmetry (0.7 s, 0%) and repetition (1.0 s, 6%) detection. Note, though, that the trend (i.e., greater costs for repetition detection) was in the same direction as for the one-handed group, and it was opposite to our prediction.

For *sensitivity* (d′), regularity-type was not significant, *F*(1, 30) = 0.04, *p* = .8, η_p_^2^ = .00. Sensitivity was similar for symmetry detection (1.47) and repetition detection (1.49). Line separation was significant, *F*(2, 60) = 14.24, *p* < .001, η_p_^2^ = .32. Post hoc Newman-Keuls analyses revealed that sensitivity to regularity detection was greater at small separations (1.76) than at medium (1.47) separations which, in turn, was greater than at large (1.21) separations (*p* < .05). In addition, the three two-way interactions were significant, though not the three-way interaction. For Regularity-type × Line separation, *F*(2, 60) = 7.25, *p* = .002, η_p_^2^ = .20, for Regularity-type × Exploration, *F*(1, 30) = 47.79, *p* < .001, η_p_^2^ = .61, and for Line separation × Exploration, *F*(2, 60) = 14.96, *p* < .001, η_p_^2^ = .33.

#### Experiments 2 and 3: Comparing Two-Handed Exploration of Midline-Aligned Stimuli (Experiment 2) Versus Across-Body Stimuli (Experiment 3) – RT and Error Analyses for Regular Trials

We conducted an ANOVA to compare the results of Experiment 2, in which 24 participants haptically explored stimuli where the axis of regularity was aligned with their body midline, and the two-handed group of Experiment 3, in which 16 participants haptically explored the same stimuli, but now oriented to be perpendicular to their body midline. All 40 participants used both of their index fingers to feel each pair of lines. There were two within-participants factors: regularity-type (symmetry or repetition) and line separation (small, medium, or large), and one between-participants factor of alignment of the axis of regularity (aligned, in Experiment 2, or across, in the two-handed group of Experiment 3).

Alignment was significant for both RT, *F*(1, 38) = 12.15, *p* < .001, η_p_^2^ = .24, and errors, *F*(1, 38) = 6.22, *p* = .02, η_p_^2^ = .14. Regularities were detected much faster and more accurately for aligned stimuli (7.9 s, 12% errors) than across stimuli (11.9 s, 19%). As detailed below, the two-way interaction of Alignment × Regularity-type, and the interaction of Alignment × Line separation, were both significant, while neither the interaction of Regularity-type × Line separation, nor the three-way interaction, was significant for either RT or for errors (all *F*s < 1.2). We presented the Results for each of these two groups separately, in the Results sections of Experiments 2 and 3, so here we will only discuss below the two significant interactions involving the factor of alignment. As before we also calculated the difference between regularity detection for the largest (100 mm) compared to the smallest (25 mm) line separation. We then repeated the above ANOVA using these (100 mm–25 mm) differences. There were no significant effects in this ANOVA.

First, the interaction of Alignment × Regularity-type was significant for both RT, *F*(1, 38) = 19.68, *p* < .001, η_p_^2^ = .34, and errors, *F*(1, 38) = 50.33, *p* < .001, η_p_^2^ = .57. Consistent with the separate group analyses already reported. Newman-Keuls analyses (*p* < .05) revealed that, for the aligned group, symmetry detection (7.2 s, 8%) was faster and more accurate than repetition detection (8.7 s, 16%), whereas the opposite was the case for the across group (13.2 s, 28% for symmetry detection; 10.7 s, 9% for repetition detection). Considering the two types of regularity separately, for symmetry detection the aligned stimuli were detected faster (by 6 s) and more accurately (by 20%) than the across stimuli, as we had predicted. For repetition detection there was a speed-accuracy trade-off: aligned stimuli were detected faster (by 2 s) but less accurately (by 7%) than across stimuli.

Second, the interaction of Alignment × Line separation was significant for RT, *F*(2, 76) = 3.49, *p* = .04, η_p_^2^ = .08, but not for errors, *F*(2, 76) = 0.47, *p* = .6, η_p_^2^ = .01. Newman-Keuls analyses (*p* < .05) revealed that the across group was slower for large separations than for medium and small separations, whereas there was no significant difference between the three separations for the aligned group.
